# Aconite

**Published:** 1885-09

**Authors:** T. C. Hunter

**Affiliations:** Wabash, Indiana


### ACONITE.

T. C. HUNTER, M. D., WABASH, INDIANA.

[Read before the Indiana Institute of Homœopathy, May 19th and 20th.]

Aconite is one of the most frequently used, least understood, and most frequently misused remedies in the materia medica. The beginner in Homœopathy has heard from his preceptors, and has read in books and journals, that it is a useful remedy in fevers. He therefore concludes that Aconite is "good for fever," and straightway gives it in all cases where fever is a prominent symptom. Our regular or allopathic friends have also learned that Aconite is "good for fever," because, he says, it is a heart and nerve sedative. He thinks that if the pulse beats less rapidly the fever is reduced, but forgets that if the pulse is reduced by force that it will react again as soon as the force is removed. He is entirely ignorant, however, of the action of Aconite upon the healthy person, as all his knowledge of medicines is gained "*ex usu in morbis.*" He knows that a knowledge of anatomy and physiology are necessary before any one can have a correct knowledge of pathology, as the latter is only sick physiology, and when he compares the condition of his patient with the healthy organism, he finds out what damage is being done. It seems strange that the thought does not suggest itself to his mind that he ought to know the effect a remedy would have upon a healthy organism before trying it on a sick one. Indeed, the idea has dawned upon the minds of a few thinkers in the regular school of medicine, but I am not aware that any systematic effort has been made to carry out the idea. It would be dangerous ground for them to tread on, as it has such a strong tendency toward Homœopathy.

Every intelligent homœopath knows that each and every remedy has symptoms peculiar to itself, which are known as "characteristic symptoms." Some of these prominent symptoms may belong to several other remedies at the same time, yet there will be such a difference in the number and arrangement of these symptoms as to lead the intelligent prescriber to the right remedy. Hahnemann says, in his preface to Aconite: "It is essential to consider the mental symptoms, to see to it that they especially are very similar if Aconite is chosen as the homœopathic remedy." This rule will, I believe, hold good with all other remedies.

We should not forget that the mind and body have very close relations, so much so that the maxim, "*Mens sana in corpore sano,*" has been in use for ages. It is then clearly our duty to mark well the effect that the body, made sick with certain medicinal substances, has upon the mind. Hahnemann says, in the same preface, that "Aconite is especially indicated when, besides thirst and an accelerated pulse, there is present an anxious impatience, a not-to-be-consoled anxiety, and an agonizing tossing about." We all know that Aconite frequently makes what seems to be almost miraculous cures of that violently painful inflammatory disease known as pleurisy; but it will be found to be only when the above mental symptoms are present, so that it does not follow that Aconite is always good for the fever of pleurisy.

If in a given case of pleurisy these mental symptoms are not present, it will be well to look further before prescribing. If the patient is "irritable and wants to be let alone," you will probably find all the other leading symptoms of the case in the pathogenesis of Bryonia.

In Aconite there are always these four conditions, viz.:

1. Thirst and an accelerated pulse.
2. Anxious impatience.
3. Inconsolable anxiety or fear.
4. Agonizing tossing about or restlessness.

You will find thirst and an accelerated pulse in many remedies. Bryonia has thirst at long intervals, drinking large draughts of water. Arsenic has continual thirst, but only taking a sip of water each time, because larger amounts are apt to cause nausea. Aconite has continual thirst, but does not desire large amounts. There is also fever with accelerated pulse without thirst in Belladonna, but all drinks are loathsome. In Helleborus we have also thirst with disgust for drink. Veratrum has thirst, but only for the coldest drinks. Chamomilla has the anxious impatience, but with it has the whining mood, while the Aconite patient complains loudly. China has inconsolable anxiety and so has Arsenic, but not so much marked by expressions of fear. The restlessness of Aconite is different from any other remedy; Arsenic has equal restlessness, but the patient wants to change beds or locations, while the Aconite patient remains in bed, but cannot remain in one position. The Rhus tox. patient moves his limbs frequently, as it gives temporary relief from pain, but he does not toss his whole body, like Aconite. It would be a waste of time to give Aconite to a quiet, non-complaining person.

Aconite has vertigo on raising the head, with vanishing of sight. Belladonna has vertigo with vanishing of sight, worse on stooping or rising from a stooping posture. Aconite has headache with increased secretion of urine, while Belladonna has headache with scanty urine. In Aconite everything tastes better but water, which is not the case with Belladonna. Both have vomiting with profuse sweat, but they differ in the amount of urine. Aconite is not likely to be of use in the vomiting stage of cholera, as suppression of urine is a leading feature of that disease. In croup we sometimes find the restless tossing about, dry cough, and difficulty of *expiration* of Aconite. The latter symptom is sometimes found in asthenia; lying on the back partially relieves the dry cough of Aconite.

The above-named four characteristics must be present if you expect the best results from the use of Aconite. They run all through its pathogenesis, and are peculiar to it alone. When they are present, all the other prominent features of the case will be found in its provings, and a mistake need not be made.

The dose must be left to the judgment of the physician. I do not doubt that good results can be had from both low and high potencies. One patient may be benefited by the crude tincture, while others may be better treated by the high or even the highest potencies. The question must be decided by the prescriber. The main point to be kept in view is the strict application of the law of similars. If low potencies aggravate the symptoms, or do not promptly benefit the patient, the observing physician will soon be led to try the higher. If high potencies fail he will try the lower, but ought never to depart from the law of similars, as only while we are obedient to law can we enjoy the largest liberty.

